# Correction: SARS-CoV-2 reinfections during the first three major COVID-19 waves in Bulgaria

**DOI:** 10.1371/journal.pone.0318435

**Published:** 2025-01-24

**Authors:** Georgi K. Marinov, Mladen Mladenov, Antoni Rangachev, Ivailo Alexiev

The Funding statement for this paper is incorrect. The correct Funding statement is as follows: A.R. would like to acknowledge the financial support of a “Petar Beron i NIE” fellowship [KP-06-D15-1] from the Bulgarian Science Fund.
