# Supplementary material for: SARS-CoV-2 reinfections during the first three major COVID-19 waves in Bulgaria
Source: PLoS One. 2022 Sep 9;17(9):e0274509. doi: 10.1371/journal.pone.0274509 (PMC9462809; doi:10.1371/journal.pone.0274509)
Supplement: Attachment — Submitted filename: 2022-05-27_response_to_reviewers.pdf [file pone.0274509.s001.pdf]

SARS-CoV-2 reinfections during the first three major COVID-19 waves in Bulgaria.
Response to reviewer comments

Reviewer 1:

> The manuscript was well written by the authors with minimum typographical errors. Authors should correct before submitting the revised manuscript by hiring a copyeditor.

We thank the reviewer for the comments.

> Even the results shown less significant. Authors should include the co-morbidity status of the patients used in this study.

> The demography of the samples collected may highlighted in detail.

We received maximally anonymized data on reinfections (this is a distinct data request from the one about general infections) from the Bulgarian Ministry of Health for the purposes of this study, thus the only demographic information contained in the dataset is the age of the reinfected individuals. We have added a figure (Figure 3C) in the revised manuscript) summarizing that distribution. We are not in possession of comorbidity information for these individuals.

> The sample volume may be increased to get a very good model of prediction.

The data presented in this manuscript is all the data available in Bulgaria; there were no more reinfections there for that time period. Thus there is no way to increase the "sample volume" as this is not an experimental, but a retrospective observational study.

Reviewer 2:

> As the title suggest the study includes data from three major waves, it would be better to include the period of three waves individually in the manuscript to give a better picture.

We agree with the reviewer in principle, however, that is already what has effectively been done in the manuscript. The great majority of reinfections occurred during the third major (Delta) wave, as this is when a variant that strongly pierced existing antibody protection first appeared while immunity had waned. Prior to that, during the first wave there were almost no reinfections (because it was the first wave), and the second (Alpha) wave came only a few months after the second, thus there were not a lot of reinfections. Analyzing the Alpha-wave reinfections alone leaves way too few examples for meaningful statistical analysis.

> However, the manuscript needs revision for language and grammar to match the publication quality.

We have carefully reviewed the manuscript for typographical errors in the revised version.
